# Incorporation of Resveratrol-Hydroxypropyl-β-Cyclodextrin Complexes into Hydrogel Formulation for Wound Treatment

**DOI:** 10.3390/gels10050346

**Published:** 2024-05-18

**Authors:** Lyubomira Radeva, Yordan Yordanov, Ivanka Spassova, Daniela Kovacheva, Ivanka Pencheva-El Tibi, Maya M. Zaharieva, Mila Kaleva, Hristo Najdenski, Petar D. Petrov, Virginia Tzankova, Krassimira Yoncheva

**Affiliations:** 1 Faculty of Pharmacy, Medical University of Sofia, 1000 Sofia, Bulgaria; 2 Institute of General and Inorganic Chemistry, Bulgarian Academy of Sciences, 1113 Sofia, Bulgaria; 3 The Stephan Angeloff Institute of Microbiology, Bulgarian Academy of Sciences, 1113 Sofia, Bulgaria; 4 Institute of Polymers, Bulgarian Academy of Sciences, 1113 Sofia, Bulgaria

**Keywords:** resveratrol, hydroxypropyl-β-cyclodextrin, complexes, hydrogel, scratch test, antimicrobial activity, skin irritation

## Abstract

Resveratrol could be applied in wound healing therapies because of its antioxidant, anti-inflammatory and antibacterial effects. However, the main limitation of resveratrol is its low aqueous solubility. In this study, resveratrol was included in hydroxypropyl-β-cyclodextrin complexes and further formulated in Pluronic F-127 hydrogels for wound treatment therapy. IR-spectroscopy and XRD analysis confirmed the successful incorporation of resveratrol into complexes. The wound-healing ability of these complexes was estimated by a scratch assay on fibroblasts, which showed a tendency for improvement of the effect of resveratrol after complexation. The antimicrobial activity of resveratrol in aqueous dispersion and in the complexes was evaluated on methicillin-resistant *Staphylococcus aureus* (MRSA), *Escherichia coli*, and *Candida albicans* strains. The results revealed a twofold decrease in the MIC and stronger inhibition of the metabolic activity of MRSA after treatment with resveratrol in the complexes compared to the suspended drug. Furthermore, the complexes were included in Pluronic hydrogel, which provided efficient drug release and appropriate viscoelastic properties. The formulated hydrogel showed excellent biocompatibility which was confirmed via skin irritation test on rabbits. In conclusion, Pluronic hydrogel containing resveratrol included in hydroxypropyl-β-cyclodextrin complexes is a promising topical formulation for further studies directed at wound therapy.

## 1. Introduction

Resveratrol is a promising polyphenol with numerous potential therapeutic applications. It exerts various pharmacological effects, such as antioxidant, anti-inflammatory, etc. [1]. In this view, resveratrol could be considered as appropriate agent for wound treatment [2]. For example, Zhou et al. observed its wound healing effect in vitro on human umbilical vein endothelial cells and in vivo on Sprague–Dawley rats [3]. The effect was probably due to upregulation of the expression of nuclear erythroid 2-related factor 2 (Nrf2) and manganese superoxide dismutase (Mn-SOD). Resveratrol also enhanced the expression of the vascular endothelial growth factor (VEGF) that promotes angiogenesis, including proliferation and migration of endothelial cells [4]. The ability of the drug to hinder inflammation via inhibiting the pathway activity of nuclear factor kappa B (NF-kB) and mitogen-activated protein kinase (MAPK) is closely related to wound healing [5]. Furthermore, resveratrol exerts antimicrobial effects against bacteria, fungi and viruses. It has the ability to affect the bacterial cell’ cycle, which results in bacteriostatic action [6] and it alters motility and the expression of virulence traits [7]. However, resveratrol is characterized by poor water solubility (0.05 mg/mL) [8]. Overcoming its low water solubility would lead to increased bioavailability, easier administration and formulation into stable dosage forms. Various approaches have been used to increase the solubility of resveratrol. For instance, it was incorporated into O/W emulsions with stevioside, where its solubility was increased 45-fold [9]. Encapsulation of resveratrol in polymeric aminoalkyl-methacrylate nanoparticles resulted in higher solubility and more pronounced hepatoprotective effects [10]. Solid dispersions prepared by spray drying resulted in almost threefold increased dissolution rate and a much higher photostability (almost sixfold compared to a resveratrol reference solution) [11]. Another approach to improving its aqueous solubility is its inclusion in cyclodextrin complexes [12].

Cyclodextrins are cyclic oligosaccharides that can form inclusion complexes due to their specific ring-shaped structure consisting of linked D-glucose units. Such inclusion complexes are distinguished by increased solubility of the active substance and, in a number of cases, by an increase in its bioavailability [13]. Cyclodextrins are known as non-toxic excipients [14]. For instance, the US Food and Drug Administration has approved 2-hydroxypropyl-β-cyclodextrin for oral and intravenous use [15]. Another important advantage of cyclodextrin complexes is that they can be included in a variety of dosage forms (tablets, eye drops, ointments, hydrogels) for different routes of administration (oral, nasal, ocular, and skin application) [16]. Dermal administration is interesting since cyclodextrins are poorly absorbed transdermally. For instance, the percutaneous absorption of hydroxypropyl-β-cyclodextrin (HP-β-CD) in aqueous solution after topical application in vivo is low (approx. 0.02% for 24 h). This fact suggests a low risk of systemic toxicity [17]. Furthermore, Loftsson discussed the potential of cyclodextrins to improve the skin penetration of poorly soluble drugs formulated in aqueous carriers [18]. One of the most commonly used cyclodextrins is β-cyclodextrin, which consists of seven glucopyranose residues linked by 1,4-glycosidic linkages. The diameter of its inner cavity makes it appropriate for the inclusion of active substances of various molecular sizes [19,20]. It has a lower hydrophilic affinity than the other representatives, which favors the formation of complexes with substances having pronounced hydrophobicity. For instance, curcumin, the solubility of which in water is less than 8 mg/L, has been incorporated into a β-cyclodextrin complex, which improved its solubility and increased its photostability [21]. HP-β-CD, a derivative of β-cyclodextrin, is characterized by lower toxicity and higher hydrophilicity [22]. Studies on antigenicity, mutagenicity and topical irritation have proven that HP-β-CD is safe. This strategy was applied by Bianchi et al., who developed a semi-solid dosage form with coumestrol [23]. The drug was included in a hydroxypropyl-β-cyclodextrin complex, which increased its solubility and enhanced its wound-healing effect [23]. Thus, the ability of cyclodextrin complexes to increase drug solubility and permeation through the skin proves their usefulness for dermal application. In this view, cyclodextrin inclusion complexes have been successfully incorporated into hydrogels to improve the dermal application of active substances [24,25,26,27,28]. Hydrogels are appropriate dosage forms for wound-healing therapies since they can provide a humid environment and protect the tissue from microorganisms’ invasion [24]. Furthermore, their high water content contributes to an easy transmission of oxygen and water vapor. The specific structure of hydrogels also allows them to retain exudates and remove water-soluble toxins [29]. Natural or synthetic polymers could be used for the production of hydrogels for wound healing. Some of the most commonly used natural polymers are alginates [30,31], chitosan [32,33], hyaluronic acid [34,35], dextran [36,37], cellulose derivatives [38,39,40] and others. The advantage of the natural polymers is that they are biodegradable, non-toxic, readily available and economically viable [41]. However, most of the time, the use of these polymers cannot lead to the desired mechanical properties and may trigger immune or inflammatory responses. In contrast, synthetic polymers could form hydrogels with desired characteristics [42]. Hydrogels have been prepared from polyethylene glycol, polyvinyl alcohol, poly(ε-caprolactone), poly(N-vinyl pyrrolidone) and others [43,44,45,46]. The triblock copolymers of poly(ethylene oxide)-b-poly(propylene oxide)-b-poly(ethylene oxide) (Pluronics) are also widely used for the preparation of thermoresponsive hydrogels because of their biocompatibility, low toxicity and good rheological properties [47]. More specifically, Pluronic F-127 is an appropriate gelling agent for hydrogels for wound healing since it could enhance collagen formation and cell attachment, resulting in improved angiogenesis [48].

The aim of the present study was to include resveratrol in hydroxypropyl-β-cyclodextrin complexes and to further formulate these complexes in hydrogels for wound treatment. The physicochemical characteristics of the complexes and their wound-healing ability (in vitro scratch test on fibroblasts) were determined. Furthermore, the antibacterial activity of pure and complex-included resveratrol against selected bacterial species (typically infecting wounds) was evaluated. The main technological and biopharmaceutical characteristics of a hydrogel formulation based on Pluronic F-127 were defined, aiming to design a topical dosage form with resveratrol.

## 2. Results and Discussion

The present study aimed to develop a topical hydrogel formulation of resveratrol considering its wound-healing potential. For this purpose, resveratrol was included in cyclodextrin complexes that were characterized with respect to their potential to improve its healing and antibacterial activity. After that, the complexes were inserted in Pluronic hydrogel, aiming to design a topical dosage form with appropriate rheological and biopharmaceutical properties. 

### 2.1. Confirrmation of Resveratrol-Hydroxypropyl-β-Cyclodextrin Complexation 

**Figure 1:**
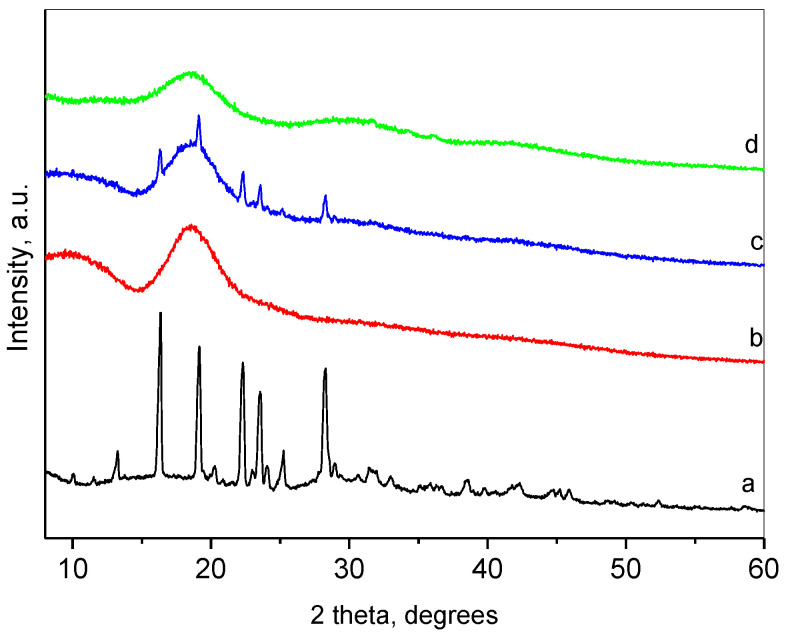
Powder XRD patterns of resveratrol (a); HP-β-CD (b); physical mixture of resveratrol and HP-β-CD; (c) and inclusion complex (d).

The complexation was performed via the solvent evaporation method, and the formation of complexes was confirmed by powder X-ray diffraction (XRD), nitrogen adsorption and FT-IR spectroscopy. Figure 1 presents the XRD patterns of pure resveratrol, HP-β-CD, their inclusion complex and physical mixture of both compounds. The XRD pattern of resveratrol was typical for a crystalline phase. The crystallographic description of this phase is Space Group P2_1_/c, and the unit cell parameters are a = 4.404(1) Å, b = 9.243(3) Å, c = 26.65(1) Å, β= 92.63(4) degree [49]. The mean coherent domain size calculated from the diffraction lines broadening was 50 ± 1 nm. The most intense peaks within the region of 6–30 degrees 2θ are presented in Table 1.

The pattern of HP-β-CD represented a broad peak centered at about 18.7°, which implied its amorphous state. The XRD of the physical mixture of resveratrol and HP-β-CD was a superposition of the patterns of the constituents. Moreover, the crystallite size of resveratrol remained unchanged, which pointed out the absence of chemical interactions between both compounds. The XRD pattern of the resveratrol-HP-β-CD inclusion complex was typical for amorphous material, with an amorphous hump centered at 18.4°. This result indicated the inclusion of resveratrol in the cavity of HP-β-CD. Thus, HP-β-CD, due to its large cavity, forms an amorphous solid with resveratrol. The method of formation of the inclusion passes through a dissolution of the resveratrol in ethanol, thus destroying its crystal structure. The inclusion of resveratrol takes place in the cavities of the HP-β-CD in a molecular form (with some specific interaction between the host and the guest). Some other authors have also reported amorphization of resveratrol upon inclusion [50,51]. 

The textural parameters of the physical mixture and the inclusion complex were determined by low-temperature nitrogen adsorption. The specific surface area, total pore volume, and average pore diameter for the physical mixture were 44 m^2^/g, 0.2 cm^3^/g, and 1.3 nm, respectively, while for the inclusion complex, they were 28 m^2^/g, 0.3 cm^3^/g, and 4.5 nm. A significant distinction in the morphological parameters could be due to the different pore structures of the physical mixture and the inclusion complex.

**Figure 2:**
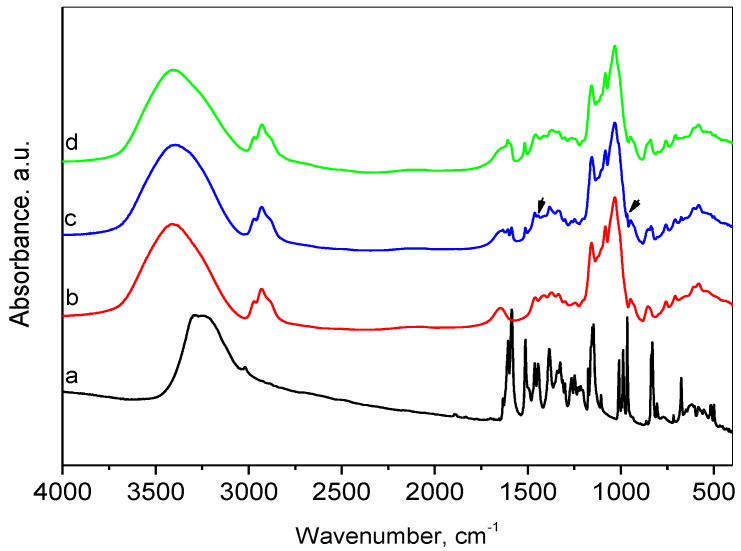
FTIR spectra of resveratrol (a), HP-β-CD (b), physical mixture of resveratrol and HP-β-CD (c), and inclusion complex (d).

The FT-IR spectroscopy was applied to investigate the inclusion of resveratrol in the HP-β-CD cavities (Figure 2). In the FTIR spectrum of resveratrol, the broad band with a maximum at about 3270 cm^−1^ is related to O–H stretching vibration of the hydroxyl groups. The low-intensity peak at 3015 cm^−1^ can be attributed to the =C–H axial stretching of bonds related to the aromatic rings. Another group of vibrations of the aromatic rings, namely, the vibrations of the C=C bonds can be associated with the presence of the bands at 1605 cm^−1^, 1591, 1514, 1464, and 1446 cm^−1^ [52]. The band at 1384 cm^−1^ was assigned to the phenolic O-H group and the band at 1148 cm^−1^ was attributed to the C–O stretching in the phenolic part. The =C–H band at 967 cm^−1^ is typical of the olefinic bond in trans-resveratrol [53]. The peaks in the region of 840–500 cm^−1^ were also related to the olefinic group. 

The spectrum of HP-β-CD presented a broad absorption band centered at 3395 cm^−1^ due to O-H stretching. Bands appearing in the region 2970–2880 cm^−1^ were due to stretching vibrations in CH_3_ (methyl in hydroxypropyl groups) and CH_2_ groups, and this at 1647 cm^−1^ was assigned to bending vibrations of water. The C–H in-plane deformation vibrations were represented by 1457 and 1372 cm^−1^ lines. The observed band at 1372 cm^−1^ is a characteristic vibration of the methyl group. The bands in the range 1200–1000 cm^−1^ with maxima at 1157, 1083, and 1032 cm^−1^ were attributed to C–O and C–O–C stretching vibrations [54,55]. The presence of glucopyranose units was confirmed by bands at 950 and 854 cm^−1^.

Comparative analysis of the spectra of resveratrol, HP-β-CD and their physical mixture confirmed the presence of both resveratrol and HP-β-CD in the mixture, as the spectrum of the physical mixture was an overlaid spectrum of both ingredients. The above-mentioned peaks, characteristic of resveratrol and HP-β-CD, were well visible. The superposition of the peaks corresponding to the OH-stretching for the physical mixture spectrum resulted in the shift of this band center to 3378 cm^−1^, which was connected to the mass ratio of the two ingredients. This observation indicated no interaction between resveratrol and HP-β-CD in the physical mixture. The comparison of the FTIR spectra of the inclusion complex and the physical mixture showed that the maximum of the characteristic absorption bands of O–H groups for the inclusion complex was shifted to 3413 cm^−1^. As the content of resveratrol in both cases was the same, the mentioned bands’ shift in the inclusion spectrum suggested the presence of interaction between both constituents. In addition, the bands observed in the resveratrol spectrum at 1446 and 967 cm^−1^ disappeared in the inclusion complex. Some authors have suggested that changes in these bands indicate that the C=C bond of the ring probably participated in the complex’s formation [56]. The interaction was also confirmed by the differences in XRD patterns and the textural parameters.

### 2.2. Wound-Healing Potential of Resveratrol in Complexes

**Figure 3:**
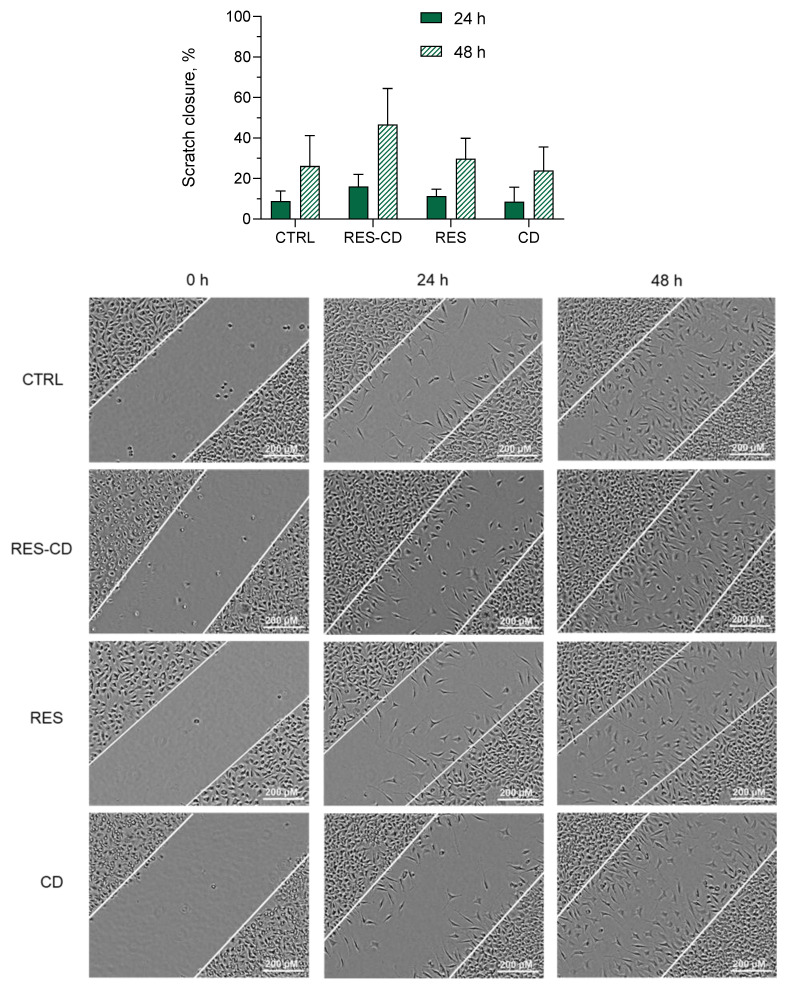
Scratch closure rate (**above**) and digital images (**below**) of the control group cells (CTRL), cells treated with resveratrol in complexes (RES-CD), standard solution of resveratrol (RES), and HP-β-CD alone (CD) at 0, 24, and 48 h.

Resveratrol is known to exhibit various activities, resulting in the wound-healing process [2,3,4,5]. Thus, the next task was to examine the possibility of improving these activities via complexation with HP-β-CD. Fibroblast cells are involved in the contraction of wounds, degradation of fibrin clots, and creation of new collagen structures and extracellular matrix [57]. Consequently, their migration is highly important for the wound-healing process [58,59]. Therefore, the effects on cell migration of a standard solution of resveratrol and resveratrol in complexes were examined via a scratch assay on L929 mouse fibroblasts. First, the inhibitory concentration of resveratrol on the selected cell line was defined (IC_50_ = 24.19 µM), aiming to perform the scratch assay with its subcytotoxic concentration. Then, the scratch assay was performed with a concentration of 10 µM of resveratrol in standard solution or in the complexes. The results revealed that the drug in the standard solution did not affect the migration of the fibroblasts (Figure 3). In comparison, resveratrol formulated into the complexes demonstrated a trend to enhance the migration of the cells. This was probably due to the increased aqueous solubility of the drug. Various studies have similarly observed an enhancement of resveratrol’s effects after increasing its hydrophilicity [60,61,62].

### 2.3. Antimicrobial Potential of Resveratrol in Complexes

**Figure 4:**
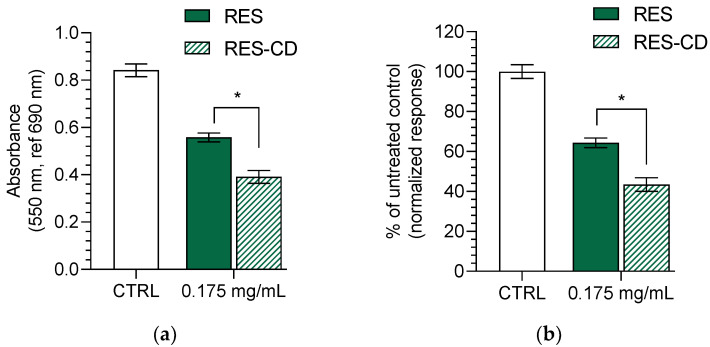
Effect of resveratrol in complexes (RES-CD) and resveratrol in aqueous suspension (RES) on the metabolic activity of methicillin-resistant *Staphylococcus aureus* (MRSA): absorbance of MRSA after treatment with both forms of resveratrol at concentration of 0.175 mg/mL (**a**); inhibition of the MRSA metabolic activity in % (**b**). * *p* < 0.05 between groups.

The bacterial and fungal colonization of wounds is a well-known process, occurring because of a favorable humid environment and a damaged skin barrier [63]. Therefore, the antimicrobial activity of resveratrol in the complex and in aqueous suspension was evaluated against two bacterial strains—one Gram-positive (methicillin-resistant *Staphylococcus aureus (MRSA)*) and one Gram-negative (*Escherichia coli*). Furthermore, their antifungal activity was examined on one fungal strain (*Candida albicans*). These strains are known to infiltrate into wounds in the early (Gram-positive) and late (Gram-negative) stages [63]. The determined minimal inhibitory concentrations (MIC) for the MRSA strain of the aqueous resveratrol suspension and resveratrol in complexes were 0.35 mg/mL and 0.175 mg/mL, respectively (Table 2). The lower MIC of resveratrol in the complexes was probably due to the increase in its aqueous solubility. Indeed, the increase in water solubility was considered a successful strategy for the improvement of the antibacterial effects of hydrophobic drugs [64,65]. The minimal bactericidal concentrations were not achieved with the maximal applied concentration of 0.35 mg/mL, which led to the conclusion that this concentration was bacteriostatic. No activity was observed for the Gram-negative bacterial strain and the fungal strain, which confirmed some previous reports. Paulo et al. discovered that a concentration higher than 400 µg/mL of resveratrol is needed to reach the MIC on *Escherichia coli* strains [6]. Moreover, two studies indicated that concentrations of 128 µg/mL [66] and 300 µg/mL [67] are not enough to reach MIC on *Candida albicans* strains. Thus, even the improved solubility of resveratrol in the complexes did not decrease its MIC for *Escherichia coli* or *Candida albicans*. However, considering the result on MRSA, we measured the metabolic activity of the bacteria treated with 0.175 mg/mL resveratrol or resveratrol in complexes. It was found that the complex of resveratrol inhibited their metabolic activity to a significantly higher degree (*p* < 0.05) (Figure 4). It is well known that the mode of action of resveratrol is related to inhibition of the electron transport chain and F0F1-ATPase [68], which reduces cellular energy production and, subsequently, bacterial proliferation. The test we used to measure the metabolic activity of the staphylococci is based on the reduction of the MTT dye by the bacterial dehydrogenases involved in electron transport. The stronger inhibition of the dehydrogenases by the complexes of resveratrol clearly revealed the enhanced antibacterial activity of the drug after its incorporation into the complexes. Although a concentration of ½ MIC of resveratrol also led to certain metabolic inhibition in the treated MRSA, there was still visible bacterial growth in the plate wells compared to those treated with the same concentration of resveratrol in complexes.

### 2.4. Incorporation of the Complexes into Hydrogel and Characterization of the Formulation

**Figure 5:**
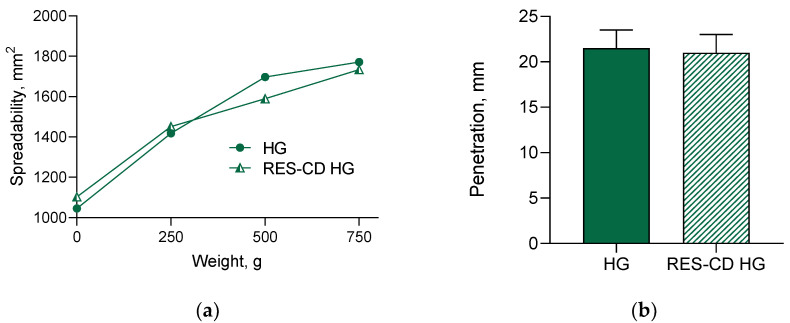
Spreadability (**a**) and penetration (**b**) of empty hydrogel (HG) and hydrogel containing complexes (RES-CD-HG).

In order to provide an appropriate application, the obtained complexes were formulated into hydrogel based on Pluronic F-127. This gelling agent was chosen since it is biocompatible and can further improve the aqueous solubility of hydrophobic drugs [69]. First, the spreadability of the formulated hydrogel was examined, since this parameter is closely related to easy administration as well as further effects of the incorporated drug [40]. In our study, the increase in the weight treatment increased the spreadability of the hydrogels, which suggests comfortable application and appropriate biopharmaceutical properties (Figure 5a). As shown, the incorporation of the complexes did not change the spreadability of the drug-loaded hydrogel. In particular, the spreadability factor for the empty hydrogel was found to be 3.81, and that for the loaded with the complexes gel was 3.77. Similarly, the results of the penetration test revealed that the empty and the loaded hydrogels possessed the same penetration ability (Figure 5b). These results prove that the incorporation of the resveratrol- HP-β-CD complexes did not influence the characteristics of the hydrogel.

**Figure 6:**
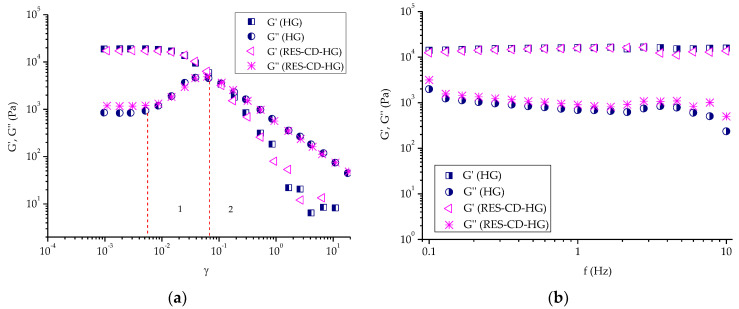
Variation in elastic (G′) and loss (G″) moduli as a function of: (**a**) shear strain (γ) and (**b**) frequency (f) for empty hydrogel (HG) and complex-containing hydrogel (RES-CD-HG).

Dynamic rheological measurements were conducted to assess the viscoelastic properties of empty and RES-CD complex-containing Pluronic F-127 hydrogels at 32 °C. Firstly, amplitude sweep tests were carried out to determine the linear visco-elastic region of each gel (Figure 6a). This region indicates the range of shear strain values (at a fixed frequency) in which the measurement can be carried out without destroying the structure of the gel. In fact, in the linear viscoelastic region (0.001 ≤ γ ≤ 0.007), the two samples exhibited similar values of the elastic (G′) and loss (G″) moduli, respectively, which means that the incorporation of the RES-CD complex did not have a notable effect on the visco-elastic properties of material. In addition, G′ was much higher that G″, indicating a hard gel structure with strong domination of the elastic over the viscous component. It should be mentioned that physical Pluronic F-127 hydrogel consists of self-assembled nanosized micelles closely packed into a three-dimensional network structure [70]. After the linear region, the G′ curves tended to drop steeply, and a crossover point (G′ = G″) was reached at γ ~ 0.1. On the other hand, the liner region G″ curves of both samples rose until reaching a distinct peak maximum (sector 1), and then slipped down (sector 2). In sector 1, G′ still dominated over G″ throughout the entire sample. However, the increase in G″ tells us that more deformation energy was lost due to internal friction during shearing. Here, a breakdown of the closely packed micellar structure started with the formation of micro breaks (cracks). The freely movable fragments (disassembled macromolecules) around the micro cracks were no longer integrated within the network and started to have internal viscous friction, thus converting the deformation energy into friction heat. With the increasing strain, the micro cracks transformed into continuous macroscopic cracks, and significant destruction of the polymer network occurred. Therefore, the viscous component dominated (G″ > G′), and the material flowed (sector 2). Apparently, Pluronic F-127 formed a physical hydrogel at 32 °C at the given concentration, and the embedded RES-CD complex did not make a significant contribution to the formation/disruption of the hydrogel network. Next, oscillation frequency sweep tests of blank and RES-CD complex-containing hydrogels were carried out in the 0.1–10 Hz range at a constant shear strain (γ = 0.002). Figure 6b shows the variation in G′ and G″ values of the two samples as a function of frequency. Overall, the two moduli were frequency-independent, and G′ > G″ was true at the specified conditions. These results confirmed that the two materials behaved as physical gels at a low level of shear strain and a temperature of 32 °C.

**Figure 7:**
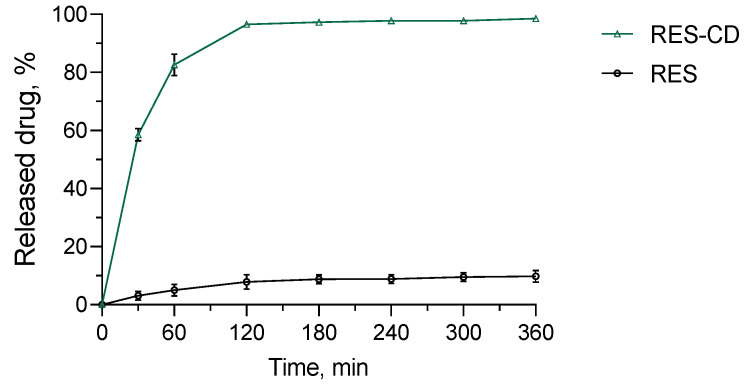
In vitro release profiles of resveratrol from the hydrogel containing its complexes (RES-CD-HG) and resveratrol from aqueous suspension (RES) in phosphate buffer (pH = 7.4).

The in vitro release test was performed in a medium with a pH value of 7.4, which represents the pH of damaged skin [71,72]. Regarding the hydrogel, almost 60% of resveratrol was released after the first 30 min, and the entire amount of the drug was released after 360 min (Figure 7). This profile was slower than the release from the complex, where, for less than 10 min, 100% of the drug was dissolved (not shown). Similar results have also been reported by other groups [73,74]. For comparison, the amount of pure drug released in the same medium after 360 min was only about 10%. Thus, it is important to note that, by incorporating resveratrol into the complexes and hydrogel, its aqueous solubility was significantly increased. The mechanism of the release from the hydrogel formulation was studied via fitting of the release data by applying four mathematical models, particularly zero-order, first-order, Higuchi and the Weibull plot. The highest correlation coefficient was defined for the Weibull function (r^2^ = 0.909) compared to the zero-order (r^2^ = 0.5072), first-order (r^2^ = 0.8079) and Higuchi model (r^2^ = 0.6866). The parameter *b*, calculated from the Weibull plot, was 0.61, suggesting Fickian diffusion in a fractal or disordered substrate [75]. In conclusion, the more efficient dissolution and the slightly slower drug release from the hydrogel could be beneficial for wound treatment.

**Figure 8:**
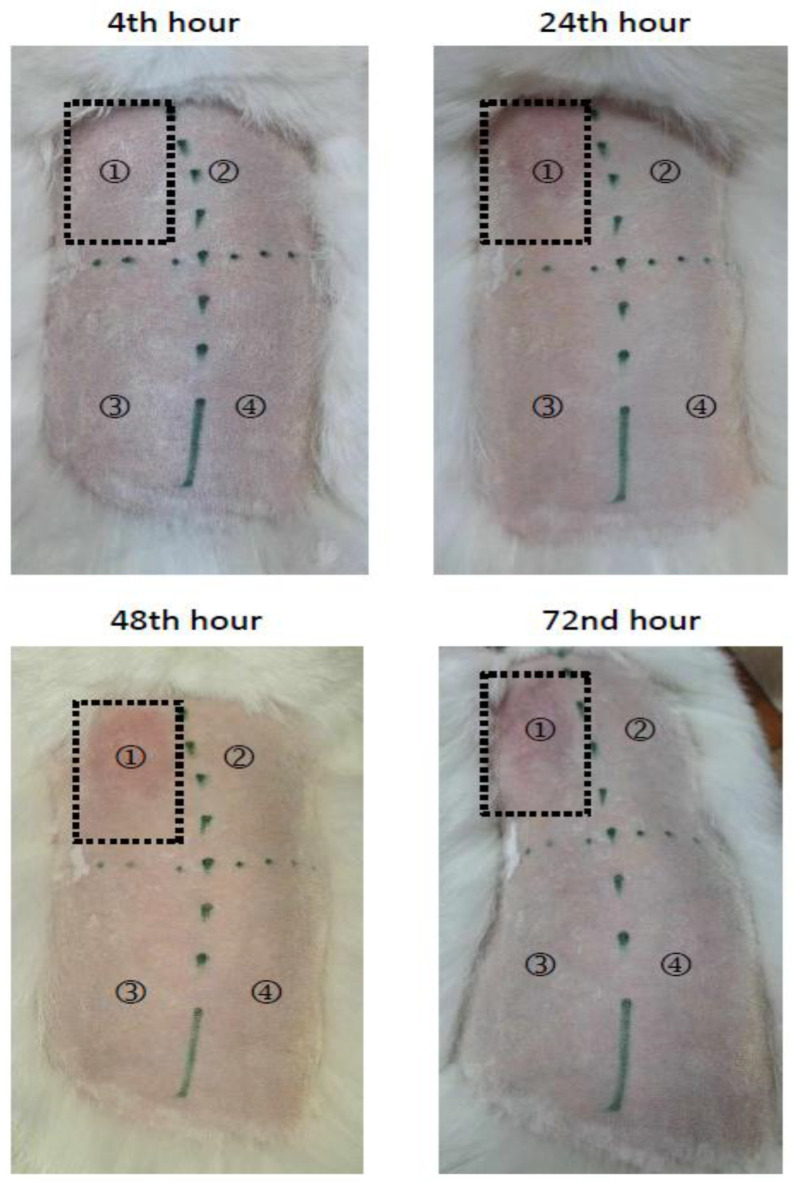
Skin irritation test of empty (HG) and complex-containing hydrogel (RES-CD-HG). Positive control (5% sodium dodecyl sulfate, site ①, empty hydrogel (site ②, complex-containing hydrogel (site ③), and the negative control (distilled water, site ④).

Finally, the formulated Pluronic hydrogel was evaluated regarding its skin compatibility via a skin irritation test on rabbit. The images comparing the treatment of rabbit skin with an empty hydrogel and a hydrogel containing resveratrol in complexes are presented in Figure 8. There was no skin reaction of erythema and/or edema after application of the empty hydrogel (Figure 8, site 2), the loaded hydrogel (Figure 8, site 3), or the negative control (distilled water, Figure 8, site 4). For comparison, irritation after treatment with a positive control (5% SDS, Figure 8, site 1) was detected. Therefore, the Primary Irritation Score (PIS) and Primary Irritation Index (PII) for the loaded hydrogel were equal to zero, whereas the values for the positive control were determined to be 2 at the 24th, 48th and 72nd hour. Thus, the study revealed that the formulated hydrogel containing the complex of resveratrol was safe for skin application.

## 3. Conclusions

Resveratrol was successfully included into hydroxypropyl-β-cyclodextrin complexes and further loaded into the Pluronic F-127 hydrogel intended for wound treatment. The complexation enhanced the wound-healing ability of resveratrol in vitro on mouse fibroblasts and decreased its minimal inhibitory concentration against methicillin-resistant *Staphylococcus aureus*. The hydrogel containing the complexes of resveratrol showed appropriate characteristics for topical application, and its biocompatibility was confirmed via a skin irritation test on rabbits. Thus, the developed formulation could be considered beneficial for wound treatment and shortening the healing time.

## 4. Materials and Methods

### 4.1. Materials

Trans-resveratrol, hydroxypropyl-β-cyclodextrin, Dulbecco’s Modified Eagle’s Medium, fetal bovine serum (FBS), and L-glutamine were purchased from Sigma Chemical Co. (Merck, Darmstadt, Germany). The murine fibroblast L929 cell line was obtained from the European Collection of Cell Cultures (ECACC, Salisbury, UK).

### 4.2. Preparation of Resveratrol-Hydroxypropyl-β-Cyclodextrin Complex

For the preparation of the complex between resveratrol and hydroxypropyl-β-cyclodextrin, a solvent evaporation method was applied [76]. The ratio between resveratrol and cyclodextrin was 1:1.5 (molar ratio). First, hydroxypropyl-β-cyclodextrin and resveratrol (3 mg) were separately dissolved in 5 mL distilled water and 1.5 mL of ethanol, respectively. After that, the solution of resveratrol was dripped to the aqueous solution of the cyclodextrin. The hydroalcoholic mixture was gently stirred (700 rpm) in order to evaporate the ethanol. Further, the obtained complexes were lyophilized.

For comparison, a physical mixture of resveratrol and hydroxypropyl-β-cyclodextrin was formulated. The physical mixture was prepared by mixing both compounds at the same ratio as that for the complexes.

### 4.3. Physicochemical Characterization of the Complexes

Powder X-ray diffraction (XRD) patterns were recorded within the 2θ region 5–60 degrees on a Bruker D8-Advance Diffractometer (Karlsruhe, Germany) equipped with X-Ray tube with CuKα-radiation (λ = 1.5418Å) and LynxEye detector. The mean coherent domain sizes (L_Vol_) were determined using Topas-version 4.2 software (Karlsruhe, Germany). The diffraction peaks were approximated using the fundamental parameters approach for the instrumental broadening implemented in the program [77].

Low-temperature nitrogen adsorption (77.4 K) was performed on a NOVA 1200e instrument. The specific surface areas were determined by the BET equation, and the total pore volumes and average pore diameters were evaluated at p/p_0_ ≈ 0.99.

The FTIR spectra were recorded in KBr pellets with a Nicolet Avatar 360 FTIR spectrometer, with accumulation of 64 scans at a spectral resolution of 2 cm^−1^.

### 4.4. Scratch Assay

The wound-healing effect of pure resveratrol or resveratrol in complexes was determined via the scratch assay on L929 fibroblasts [78,79]. Briefly, the cells were seeded in 24-well plates at a 10^5^ cells/mL. They were incubated for 24 h in DMEM medium supplemented with 10% FBS at standard conditions (37 °C, 5% CO_2_, and high humidity in Esco CelCulture^®^ CO₂ Incubator, CCL-170B-8-IVF, Esco Micro Pte. Ltd., Singapore) in order to obtain a confluent monolayer. Thereafter, the wells were scratched with a sterile pipette tip (200 µL), and the cells were washed twice with the medium. Then, it was aspirated, and medium without fetal bovine serum was added in order to exclude the influence of proliferation. The fibroblasts were treated with resveratrol in ethanol and resveratrol in complexes at a 10 µM concentration, as well as hydroxypropyl-β-cyclodextrin in a concentration corresponding to the complex. The cells were photographed with an inverted light microscope (Optika XDS-2) and digital camera (Optikam Pro 8LT—4083.18LT) at 0, 24, and 48 h. ImageJ software (version 1.54g) was used for analysis of the images [80]. The wound-healing ability was determined via calculating the scratch closure rate as a percentage, according to the following expression:

Scratch closure rate (%) = 100−A_s_ × 100/A_0_, (1)

where A_0_ corresponds to the scratch area at 0 h and A_s_ corresponds to the scratch area at 24 h or 48 h.

### 4.5. Antimicrobial Studies

The antimicrobial activity of resveratrol in the complexes and aqueous suspension of resveratrol was tested on one Gram-positive bacterial strain, methicillin-resistant *Staphylococcus aureus* (#NBIMCC 8327, MRSA, National Bulgarian Collection of Industrial Microorganisms and Cell Cultures); one Gram-negative bacterial strain, *Escherichia coli* (ATCC^®^ 35218^TM^, Manassas, VA, USA); and one fungal strain, Candida albicans (CBS 562, Utrecht, The Netherlands). MRSA and *Escherihia coli* were maintained in Mueller Hinton agar (MHA, #CM0337, Thermo Scientific-Oxoid, Hampshire, UK) and Mueller Hinton broth (MHB, #M0405B, Thermo Scientific-Oxoid, Hampshire, UK). *Candida albicans* was grown in brain heart infusion broth (#M210, Himedia, Mumbai, India). The broth microdilution assay was performed in MHB and BHI according to ISO 20776/1-2006 [81], which represents the broth microdilution test.

The metabolic activity of MRSA was determined after treatment with both forms of resveratrol at a concentration of 0.175 mg/mL (MIC of resveratrol in complexes). The test was performed as described in a previously published paper by Yoncheva et al. [64]. Briefly, 10 µL of MTT solution (5 mg/mL in PBS) were added to each well, and the plate was incubated for 120 min at 37 °C. The reaction product formazan was dissolved with an equivalent volume of organic solvent (2-propranol with 5% formic acid). The absorbance was measured at 550 nm against blank solution and a 690 nm reference filter on a microplate reader (Lx800, Bio-Tek Instruments Inc., Winooski, VT, USA). The blank solution contained the growth medium used for each bacterial/fungal strain, MTT solution, and the organic solvent. The metabolic activity was calculated as a % of the untreated control.

### 4.6. Incorporation of Resveratrol-Hydroxypropyl-β-Cyclodextrin Complexes in Hydrogel 

For the preparation of the hydrogel formulation, Pluronic F-127 was used as a gelling agent. Briefly, Pluronic F-127 (25 wt%) was dispersed in the aqueous solution containing the resveratrol complexes. The resulting dispersion was left in a refrigerator (4 °C) overnight in order to homogenize the gel. The empty hydrogel was prepared according to the same procedure, using distilled water as a medium.

### 4.7. Characterization of the Hydrogel Formulation

Dynamic rheological measurements of the hydrogels were carried out with a HAAKE MARS 60 rheometer in controlled deformation mode using a parallel plate sensor system (top plate diameter = 20 mm; gap = 1 mm). The elastic (G′) modulus was determined at 32 °C and constant deformation (γ = 0.01) in the 0.1–10 Hz frequency range.

The parallel-plate method [82,83] was applied in order to determine the spreadability of the empty hydrogel (HG) and hydrogel containing resveratrol in complexes (RES-CD-HG). Briefly, 1 g of the hydrogel (empty or containing the complexes) was placed between two glass plates (20 × 20 cm), and exact weights of 250, 500, and 750 g were placed onto the upper glass for 3 min. The diameter of the sample between the plates (d) was measured, and its value was used for the calculation of the spreadability and spreadability factor:

S = d^2^ × π/4, (2)

where S is the spreadability of the sample (mm^2^) and d is the diameter of the sample (mm).

Sf = S/P,(3)

where Sf is the spreadability factor of the sample (mm^2^/g) and P is the charging weight (g).

The pharmacopoeial penetrometry test was used for the determination of the consistency of the hydrogels. Sufficient amounts of the gel samples were prepared and, immediately after gelation, were stored in the test container for 24 h at 25 ± 0.5 °C prior to testing. The gravity-driven penetrating object was released for 5 s. The penetration depth was measured and presented in millimeters.

### 4.8. In Vitro Drug Release Tests

The in vitro release tests were conducted in a phosphate buffer (pH of 7.4) containing 10% ethanol under 32 °C and gentle shaking (IKA Labortechnik HS-B20, Staufen, Germany). Briefly, 2.5 g of the hydrogel (containing 1.16 mg of resveratrol) was placed in 40 mL of the medium. For comparison, an aqueous suspension of resveratrol was introduced into a dialysis membrane and placed in the same medium. At predetermined time intervals, 2 mL of the acceptor phase was taken. Aliquot amounts of fresh medium were returned in order to maintain sink conditions. The concentration of the released drug was determined via high-performance liquid chromatography (HPLC, Thermo Scientific UltiMate Dionex 3000 SD, Chromeleon 7.2 SR3 Systems, Thermo Fisher Scientific, Waltham, MA, USA). Briefly, the system consisted of a Phenomenex C18 Column—Luna (250 mm × 4.60 mm, particle size 5 μm) and an isocratic mobile phase containing methanol:water:anhydrous acetic acid at a ratio of 52:48:0.05 (*v*/*v*/*v*) and a flow rate of 1.0 mL/min [84]. The detection wavelength was set at 303 nm. The column and the HPLC system were kept at 25 °C ± 1 °C.

### 4.9. Skin Irritation Test

The dermal irritation potential of the empty and complex-loaded hydrogel were evaluated following the animal irritation test protocol 6.3 in ISO 10993-10 [85]. The approval for performing an irritation test with rabbits was issued by the National Commission of Animal Ethics, Bulgarian Food Safety Agency at the Ministry of Agriculture and Food (Nr. 232/2020 valid to 11 April 2024). As required in the standard, the test was carried out with three healthy, young New Zealand albino rabbits with intact skin in the animal house of the Stephan Angeloff Institute of Microbiology (Reg. Nr. 1113-0005). The acclimatization of the experimental animals followed the rules of ISO 10993-2 [86] and Ordinance Nr. 20 (State Gazette of Bulgaria, Nr. 87, 9 November 2012).

### 4.10. Statistical Analysis

The experiments were performed in triplicate, and the results are expressed as mean values ± SD. The data were statistically analyzed using GraphPadPrism, version 8 (GraphPad Software, San Diego, CA, USA). One-way ANOVA with Dunnett’s multiple comparison post-test and multiple *t*-tests, followed by the Holm–Sidak post-test, were performed. 

## Figures and Tables

**Table 1:** Miller indices and 2θ positions of the most intense peaks of resveratrol.

hkl	(002)	(004)	(014)	(020)	(015)	(022)	(016)	(110)	(1-1-2)
2θ°	6.63	13.28	16.39	19.18	19.21	20.32	22.19	22.36	23.08
hkl	(024)	(112)	(1-1-3)	(017)	(121)	(018)	(122)	(124)	
2θ°	23.41	23.61	24.14	25.29	28.29	28.47	29.0	31.49	

**Table 2:** Minimal inhibitory concentrations (MIC) of resveratrol in complexes (RES-CD) and resveratrol in aqueous suspension (RES) estimated on two bacterial strains and one fungal strain.

Bacterial Strain	Minimal Inhibitory Concentration (mg/mL)	
	RES	RES-CD
Methicillin-resistant *Staphylococcus aureus* (MRSA), #NBIMCC 8327	0.35	0.175
*Escherichia coli*, ATCC^®^ 35218T	>0.35	>0.35
*Candida albicans*, CBS 562	>0.35	>0.35

## Data Availability

Data are contained within the article.
